# Risk Factors for Sensorineural Hearing Loss in Neonatal Hyperbilirubinemia

**Published:** 2018-07

**Authors:** Hassan Boskabadi, Maryam Zakerihamidi, Ali Moradi, Mehdi Bakhshaee

**Affiliations:** 1 *Department of Pediatrics, School of Medicine, Mashhad University of Medical Sciences, Mashhad, Iran.*; 2 *Department of Midwifery, School of Medicine, Islamic Azad University, Tonekabon Branch, Tonekabon, Iran.*; 3 *Orthopedic Research Centre, Ghaem Hospital, Mashhad University of Medical Sciences, Mashhad, Iran.*; 4 *Sinus and Surgical Endoscopic Research Center, Faculty of Medicine, Mashhad University of Medical Sciences, Mashhad, Iran.*

**Keywords:** Hearing Loss, Jaundice, Newborn, Risk factors, Sensorineural

## Abstract

**Introduction:**

Hyperbilirubinemia is a common neonatal problem with toxic effects on the nervous system that can cause hearing impairment. This study was conducted to assess the risk factors for sensorineural hearing loss and other coexisting problems in icteric infants.

**Materials and Methods:**

In a case-control study, 200 term infants with bilirubin levels higher than 20 mg/dl admitted to the neonatal intensive care unit of Ghaem Hospital, Mashhad during 2007–2015 were investigated. Profiles of infants with hearing impairment (n=60) were compared with those of icteric newborns with normal hearing (140 newborns) as the control group. After confirming the clinical diagnosis of jaundice by laboratory findings, a validated questionnaire containing mother and infant profiles were used for data collection. The auditory brainstem response test was used for assessment of infant hearing status after discharge.

**Results:**

Sensorineural hearing loss among infants with severe hyperbilirubinemia was found to be 4.8%. Serum total bilirubin (P=0.001), creatinine levels (P=0.002), direct Coombs test results (P=0.001), etiology (P=0.000) and treatment for jaundice (P=0.000), eye movement disorders (P=0.001), opisthotonos (P=0.001), and microcephaly (P=0.001) were found to be significantly different between the two groups (P<0.005). The prognostic predictability of sensorineural hearing loss based on total bilirubin level was found to be 82%.

**Conclusion:**

Hearing impairment occurs about 10–50 times more frequently in neonates with severe jaundice. Total bilirubin level has the highest predictability for infant hearing status. Blood group and Rhesus (Rh) incompatibilities between mother and child and G6PD deficiency are important known causes for hearing impairment due to jaundice.

## Introduction

Neonatal jaundice is the most common cause of hospitalization in the first month after birth (1,2). Bilirubin levels exceeding beyond the 95% percentile in 8–11% of newborns necessitates further examination and treatment (2), otherwise, serious complications such as kernicterus can lead to lifelong disabilities (3,4). Finding an appropriate approach through early diagnosis, treatment, and follow-up of icteric infants has always been a major challenge in neonatology. Prevention, early diagnosis, and appropriate treatment can decrease the rate of potential complications. Despite recent progress in jaundice care, bilirubin toxicity remains one of the major challenges in neonatal care (5). Auditory pathways are the sensitive parts of the nervous system to the toxic effects of bilirubin. Neonatal jaundice is one of the causes of early sensorineural hearing loss in developing countries, and increased blood indirect bilirubin can cross the blood-brain barrier and deposit in the auditory ventricular nucleus cells (6).

Risk factors for hearing loss vary at different ages. A family history of hearing loss, congenital infections, auditory-pharyngeal disorders, meningitis, ototoxic medications, and bilirubin levels higher than 20 mg/dl are involved in hearing loss in children under 2.5 years old (7,8). The annual global economic burden of hearing impairments is estimated to be 150 million USD, and the World Health Organization aims to prevent hearing loss by reducing the diagnosis age to the time of birth (9). Thirty percent of children with learning disabilities suffer from hearing loss. In most cases, the detection of hearing loss in children is delayed, and the appropriate speech-learning time is missed, while 90% of deaf children have parents with normal hearing status (10). Since early detection and treatment of hearing loss during the first 6 months of infancy has the best impact on language development, performing infantile screening tests for detection of hearing loss is also necessary (11,12).

The otoacoustic emission (OAE) test is one of the routine after-birth screening procedures for risk-factor-free infants (12,13). 

The auditory brainstem response (ABR) is another test used to detect hearing loss and neural type in particular (11), and has the efficiency and sensitivity required for infants with hyperbilirubinemia (14,15). Several studies have investigated the relationship between hyperbilirubinemia and hearing impairments. Kernicterus has been reported as the most common cause of acquired deafness (15), and the risk of hearing loss has been shown to increase with prolonged exposure to hyperbilirubinemia (16). The status of ABR in infants with high indirect bilirubin has shown that increased blood indirect bilirubin (>20 mg/dl) causes hearing impairment in newborns (17). As most previous studies have been concerned about the hearing status of icteric newborns, the risk factors of infantile hearing impairment and its accompaniment with other coexisting problems in icteric infants have not been properly investigated. The aim of the current work is to study the risk factors of hearing loss and coexisting problems in newborns with jaundice.

## Materials and Methods

In this case-control study conducted between 2007 and 2015, 200 term infants with bilirubin levels higher than 20 mg/dl admitted to the neonatal intensive care unit of Ghaem Hospital, Mashhad were investigated. Profiles of infants with hearing impairment (n=60) were compared with those of icteric newborns with normal hearing (140 newborns) as the control group. The exclusion criteria included multiple anomalies, chromosomal diseases, history of hearing impairment in the family members, and asphyxia. 

Convenience sampling was done from all icteric neonates admitted to Ghaem Hospital, Mashhad. Data collection was performed using a questionnaire. The validity and reliability of the questionnaire was confirmed according to reliable sources and the judgment of five experts using alpha Cronbach’s reliability test (r=0.8). The questionnaire consisted of maternal and infant demographic data as well as the Denver II developmental screening test. Patients were evaluated after obtaining their parents' consent. The study was performed after ethical approval from the Vice Chancellor for Research, Mashhad University of Medical Sciences.

Complete physical examination of infants as well as medical history, age, gender, birth weight, gestational age, and Apgar score were recorded, together with the mother's age and blood type. In addition, the infant’s bilirubin, hematocrit, direct and indirect Coombs levels, reticulocytes count, glucose-6-phosphate dehydrogenase (G6PD) enzyme levels, and both the mother's and infant's blood type and complete blood cell count were determined. ABO incompatibility was suspected when the mother's blood group was type O and the baby's blood group was either A or B, with at least two of the following conditions: jaundice on the first day, positive direct Coombs test, microspherocytosis in the peripheral blood or positive indirect Coombs test (5,19). 

In the absence of Rhesus (Rh) or ABO incompatibility, but a positive direct Coombs test, the incompatibilities were considered as a sub-group (19). The point fluorescence method was used for G6PD enzyme measurement, with an enzyme activity level below 30% considered inadequate. The criteria for confirmation of infection included positive urine or/and blood culture. The urine sample was aspirated through suprapubic puncture. The culture was considered positive if any colony-forming unit of a single pathogen was isolated. The urine samples were microscopically examined for leukocyturia (>5 leukocytes/high power field) and bacteriuria. 

***Hearing assessment***

The hearing status of the neonates was investigated using the ABR method after discharge. ABR is a non-invasive method for early detection of impaired hearing pathways. The efficiency of the test increases with prolonged exposure to hyperbilirubinemia, even after effective treatment. ABR is also capable of detecting subclinical encephalopathy before the manifestation of signs and symptoms of kernicterus. ABR test impairment is usually transient in most patients and improves with rapid and effective treatment. Thus, performing serial ABR tests can be a useful, non-invasive and necessary technique to diagnose disorders ranging from secondary neurodevelopmental disorders to hyperbilirubinemia (20). The ABR test was performed using a GSI device 2012 (Denmark-America) in the frequency range 1–3 kHz with a click-type stimulant and the following parameters: polarity=35 dB alternative unilateral, frequency=16–24 pulse/s, trials=3,000, and analysis time=10 ms. The evoked electrical responses were recorded by electrodes attached to the scalp skin and the mastoid process during the first 10 ms after auditory stimulation (21). Among the 5–7 waves recorded in ABR, waves I, III and V can be consistently achieved for all age groups, while waves II and IV are seen less consistently. Sensorineural hearing impairment is defined by disappearance or delay of waves and reduced amplitude of wave V (22). Infant follow-up assessments were conducted at 6 and 12 months of age using the Denver II developmental screening test as well as the complications assessment (i.e. jaundice-induced visual, hearing, and motor disorders). The Denver II developmental test evaluates children’s personal-social, fine motor, language, and gross motor development. With any problems in each of the aspects of the test, the condition is considered as a developmental delay. Impairment of only one, two, or three or more aspects are considered as mild, moderate, and severe developmental delays, respectively (23). 

***Statistical analyses***

SPSS (16.5) software was used for data analysis. Data are expressed as the mean ± standard deviation (SD). The Student t-test or Chi-square test was used to analyze the relationships between variables with normal distribution and nominal scale, respectively. A P<0.05 was considered statistically significant.

## Results

**Fig 1:**
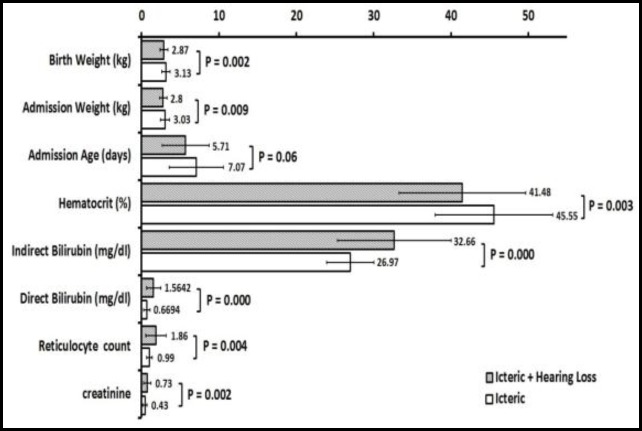
Comparison between the clinical and laboratory parameters in icteric newborns with sensorineural hearing loss and icteric infants with normal hearing

Of 200 icteric neonates who participated in this study, 60 newborns suffered from sensorineural hearing loss (case group), whose characteristics were compared with 140 infants with a history of a bilirubin level of over 20 mg/dl having a normal hearing status (control group). Of the 200 icteric infants studied, 58% were males and 42% were females. The average referral ages of icteric infants in the control and test groups were 7.07 ± 3.53 and 5.71 ± 3.02 days, respectively. The mean birth weights of infants in the control and case groups were 3.13 ± 0.54 and 2.87 ± 0.51 kg, respectively. The mean gestational ages of infants in the control and case groups were 39.1 ± 0.86 and 38.6 ± 1.31 weeks, respectively. The mean Apgar scores of infants in the control and case groups were 8.9 ± 1.11 and 8.6 ± 1.31, respectively. Other characteristics of the studied infants are given in Figure 1.

There was no significant difference between the two study groups regarding the following factors: parity (P<0.630), maternal age (P<0.725), T4 (P<0.132), thyroid-stimulating hormone (TSH) (P<0.413), or blood urea nitrogen (BUN) (P<0.580). However, the differences were statistically significant between the two groups in the following parameters: birth weight (P<0.002), admission weight (P<0.009), age (P<0.06), direct bilirubin (P<0.001), total bilirubin (P<0.001), hematocrit (P<0.003), reticulocyte count (P<0.004), and creatinine (P<0.002) (Fig. 1).

**Fig 2:**
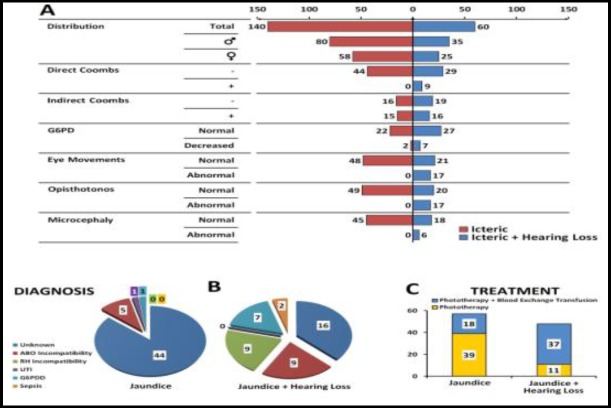
Comparison of some infant variables between the icteric newborns with and without sensorineural hearing loss. A) Distribution of clinical and laboratory results between the two groups, B) Comparison of the diagnosis of coexisting problems between the two groups, and C) Comparison of the major treatment strategies between the two groups

No significant statistical association was found between the infant's hearing status and sex (P<0.962). The direct Coombs test result was significantly associated with hearing status (P<0.001), meaning that the newborns with sensorineural hearing loss had the highest value of positive direct Coombs results. However, no significant association was seen between hearing loss and indirect Coombs results (P<0.101). The rate of G6PD deficiency was significantly different between the study and control groups (P<0.04). The etiology of jaundice in infants with sensorineural hearing loss was one of the following conditions: unknown (16 cases), blood group (ABO) and Rh incompatibility (nine cases each), G6PD deficiency (seven cases) and sepsis (two cases); while in icteric infants with normal hearing status, the etiology of jaundice was as follows: unknown (44 cases), ABO incompatibility (five cases), urinary tract infection (one case), G6PD deficiency (case case), and sepsis (two cases). Significant differences were seen in abnormal eye movements (P<0.001), opisthotonos (P<0.001), and microcephaly (P<0.001) between the two groups, indicating that eye movement disorder, opisthotonos and microcephaly were seen with a higher rate in jaundiced infants with sensorineural hearing loss (Fig.2). The paired t-test showed that admission weight was lower than birth weight (P<0.001), indicative of significant weight loss during the birth to admission period.

**Fig 3:**
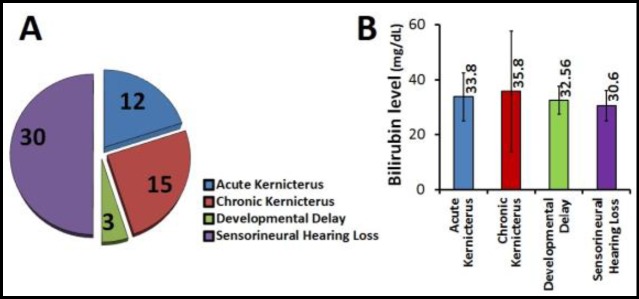
A) Distribution of final outcomes of infants with hearing impairment in long term, B) Comparison of mean bilirubin levels in infants with the final outcomes

Long-term final outcomes of infants with hearing impairment at 12 months of age are shown in Figure 3A. Infants with chronic kernicterus had the highest levels of total bilirubin (Fig. 3B).

**Fig 4:**
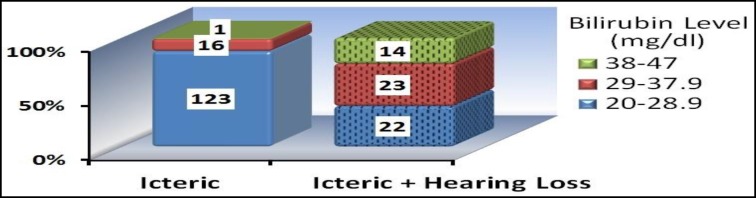
Comparison between the control and case groups at different levels of bilirubin Data analysis based on regression models showed that the bilirubin levels have a high predictive power (82%) for determining the prognosis of sensorineural hearing loss in icteric newborns (Table 1).

Comparison of icteric infants with and without normal hearing based on three various levels of bilirubin revealed a significant relationship between the bilirubin levels among the three groups (P< 0.001) (Fig.4).

**Table 1:** Comparison of combined parameters of bilirubin levels, diagnosis, and treatment in predicting the risk of hearing loss in icteric infants

**Diagnostic methods**	**-2 log likelihood**	**Cox & Snell R square**	**Nagelkerke R square**	**Hosmer & Lemeshow test**		**Predicted percentage correct**
Bilirubin levels + cause of jaundice + treatment method	79.692	0.412	0.551	0.128	78.7	
Bilirubin levels + cause of jaundice	82.036	0.397	0.531	0.402	75.5	
Bilirubin levels	197.434	0.209	0.296	0.001	82	

## Discussion

According to our study results, the risk of hearing impairment in icteric infants with a bilirubin level higher than 20 mg/dl was 10–50 times higher. Sensorineural hearing loss was found to be related to severity of hyperbilirubinemia, cause of jaundice, and treatment approach. In addition, an increased rate of complications including microcephaly, abnormal eye movements, and developmental delay was seen in icteric infants with hearing loss. While other studies have reported a diverse range of incidence rates for abnormal ABR test results among infants with hyperbilirubinemia (17,19,23-27), our findings showed an incidence rate of 4.8%. However, hyperbilirubinemia requiring blood exchange transfusion can damage the hearing system and disrupt hearing tests even without developing kernicterus (28). The mechanisms underlying bilirubin neurotoxicity have not yet been elucidated. Also, it is not clear how only some of the infants with a certain level of bilirubin develop hearing loss or neurological damage (29). The lower incidence of hearing impairment in our study compared with similar studies may be due to different methodologies and sample sizes. On the other hand, while most other studies performed the hearing tests during jaundice, the infants in the current study were examined for hearing status at the first month after developing jaundice. Clearly, the rate of hearing impairment is expected to be higher at the acute phase of jaundice compared with the post-treatment (phototherapy, blood exchange transfusion) period when many cases of hearing loss have been alleviated and only persistent cases remain. The final outcomes of icteric newborns with hearing impairment included acute kernicterus (6%), chronic kernicterus (7.5%), developmental delay (1.5%), and only sensorineural hearing loss (15%). In a similar study, pre-treatment abnormal ABR was reported as 28.3%, but decreased to 8.3% in post-treatment infants, lasting up to 3 months of age (19). In a different study, 74.3% of infants ultimately developed normal hearing, while 25.7% were still suffering from hearing loss (30).

Our findings indicating a higher risk of developing hearing loss with increased severity of hyperbilirubinemia confirm the results of previous studies (17,19,31).

The most common causes of jaundice among infants with sensorineural hearing loss in our study include unknown causes, ABO and Rh incompatibility, G6PD deficiency, and sepsis. The most common causes of hyper-bilirubinemia in infants with normal hearing were unknown, ABO incompatibility, urinary tract infections, G6PD deficiency, and sepsis. Idiopathic causes (30%), ABO incompatibility (18%), Rh incompatibility (14.8%), G6PD deficiency (12.6%), and sepsis (3.3%) have been described in other studies as the most common predisposing factors for jaundice complications, including hearing loss disorders (32).Routine ABO and Rh tests are commonly performed for mothers in our medical center. In the case of a Rh-negative mother, the infant's umbilical cord blood is tested for ABO, Rh, and direct Coombs. However, there is no definite program for outpatient follow-up and longer screening to ensure the absence of jaundice. Hence, Rh incompatibility is still the second most common known cause of hearing disorder in our study.

G6PD deficiency was the third etiology leading to hearing impairments. As a result of lack of a proper screening system for the G6PD enzyme, as well as the parents' lack of knowledge, the affected newborns are usually presented late to the hospital and have already developed higher jaundice complications, including hearing impairment.

According to this study, more icteric infants with sensorineural hearing loss have undergone a combination of phototherapy and blood exchange transfusions, which is in line with other similar studies (24,33). Low birth weight, exchange transfusion due to hyperbilirubinemia and low Apgar score in the first minute of birth have been reported as the most important risk factors for hearing loss in newborns (34). Exchange transfusion usually has a close relationship with the severity of hyperbilirubinemia, jaundice etiology, and clinical symptoms in the newborn. However, the duration of exposure to elevated bilirubin levels in our newborns was higher due to late referral, which increases the risk of complications, even despite blood transfusions.

Icteric infants with sensorineural hearing loss in this study had lower admission ages and higher bilirubin levels, while ABR has been shown to be independent of age, weight, bilirubin levels, and blood group in other studies (28). The impact of the duration of hyperbilirubinemia as well as its amplitude might be the reason for our different findings. In addition, infants with kernicterus might have been visited sooner due to its symptomatic manifestations. According to our study results, 17% of infants with sensorineural hearing disorder had abnormal eye movements, while 17% and 6% suffered from abnormal opisthotonos and microcephaly, respectively. In other studies, hyperbilirubinemia has been reported to cause visual disturbances, mobility problems, cerebral palsy and seizures in addition to hearing losses (35,36). Results over a long-term, 3-year follow-up in icteric children has shown other complications including the full neurological syndrome of bilateral choreoathetosis with involuntary muscular spasms, extrapyramidal symptoms, seizures, mental retardation, dysarthria, hearing loss in high frequencies, strabismus, and abnormal upward eye movements (1).

## Conclusion

In this study, the rate of hearing loss sensorineural was equal to 4.76%. In addition, icteric infants with sensorineural hearing impairment compared to icteric babies with normal hearing status are at risk of low birth weight, the need for blood exchange transfusions, eye movement disorder, opisthotonos, and microcephaly disorders. Blood group and Rh incompatibilities and G6PD deficiency are the important known causes for hearing impairment due to jaundice. 

Bilirubin levels have a high predictive power (82%) for prognosis of sensorineural hearing disorders in icteric neonates. Higher levels of bilirubin are associated with a greater risk of developing hearing losses. Over 90% of icteric infants with bilirubin levels of 38-47 mg/dl develop hearing impairments. Great emphasis must be laid upon screening the hearing status of infants with hyperbilirubinemia due to the importance of hearing performance in child learning processes.
